# Supplementary material for: Cortical dynamics in hand/forelimb S1 and M1 evoked by brief photostimulation of the mouse’s hand
Source: bioRxiv. 2025 Mar 13:2024.12.02.626335. Originally published 2024 Dec 2. Preprint. [Version 2] doi: 10.1101/2024.12.02.626335 (PMC11642753; doi:10.1101/2024.12.02.626335)
Supplement: 1 [file NIHPP2024.12.02.626335v2-supplement-1.pdf]

bioRxiv preprint doi: https://doi.org/10.1101/2024.12.02.626335; this version posted March 13, 2025. The copyright holder for this preprint (which was not certified by peer review) is the author/funder, who has granted bioRxiv a license to display the preprint in perpetuity. It is made available under aCC-BY 4.0 International license.

Figure 1 – video 1

Top left: example video of a mouse during phototactile stimulation, recorded at 1000 frames per second and downsampled to 30 frames per second for display. Approximate LED onset times are indicated (exact onset times cannot be shown as the frame period is longer than the LED illumination duration). The ROI for assessing digit movement is indicated by a green rectangle. **Top right:** difference in pixel value from the baseline (average of the 100 ms prior to each flash) for the same, pseudocolored so that negative values are shown in blue and positive values in red. **Bottom:** trace showing the average change in pixel value over the indicated ROI for each frame. The currently displayed video frame is indicated with a red circle. The duration of LED illumination for each flash is indicated in blue. Note the artifact due to the video camera capturing the light from the LED, which was excluded from analysis.

bioRxiv preprint doi: https://doi.org/10.1101/2024.12.02.626335; this version posted March 13, 2025. The copyright holder for this preprint (which was not certified by peer review) is the author/funder, who has granted bioRxiv a license to display the preprint in perpetuity. It is made available under aCC-BY 4.0 International license.

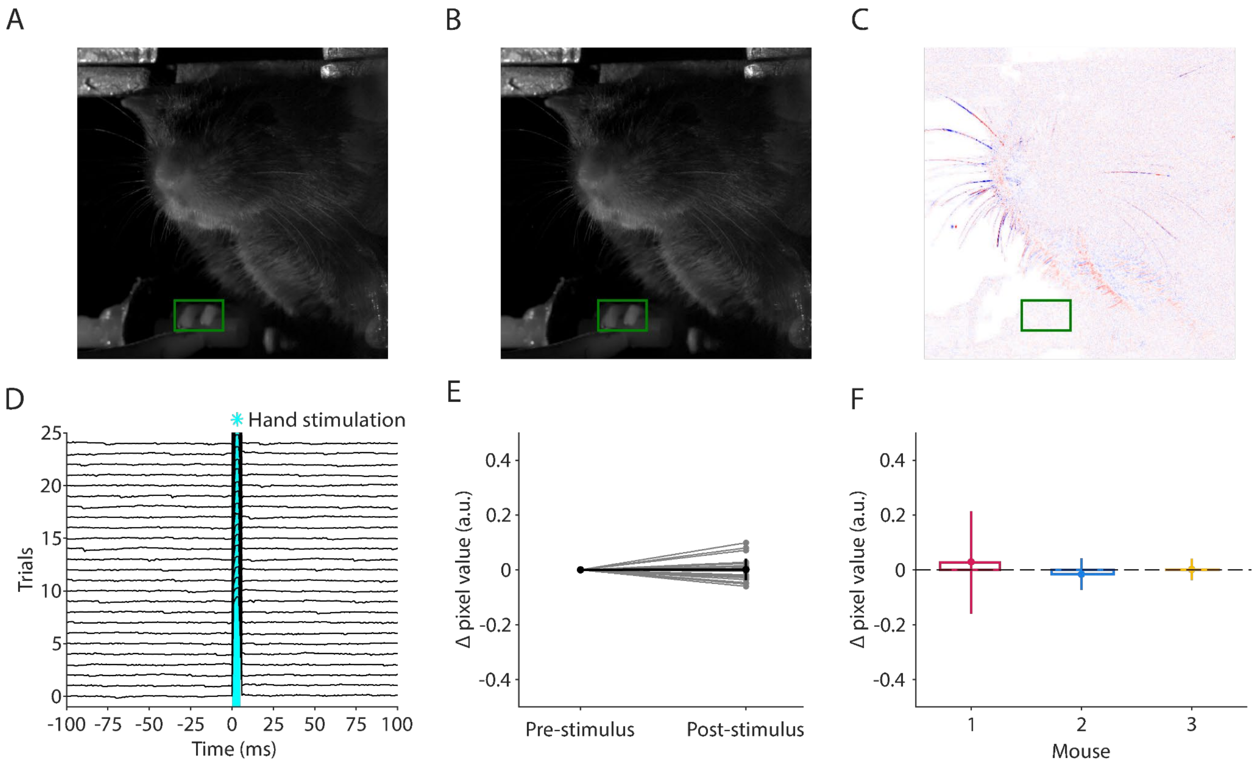

Figure 1 - figure supplement 1. Optogenetic stimulation of mechanosensory afferents in the mouse's hand does not evoke forelimb movements

(A) Average video frame during the baseline period from 100 to 0 ms before stimulus onset for one example photostimulation trial for one example mouse. The ROI for detecting hand movements is indicated in green.

(B) Example video frame from the post-stimulus interval for the same stimulus and mouse in **(A)**.

(C) Difference image between the frames in **(A)** and **(B).**

(D) Average change in pixel values from baseline over the green ROI for all trials for the same mouse depicted in **(A-C)**. Blue shaded area represents the time when the LED was on (5 ms duration, 5 mW light intensity at the fiber tip, 910 µm core diameter).

(E) Average change in pixel values over the green ROI in the period from -100 to 0 ms before the stimulus ('Pre-stimulus') and from 6 ms to 100 ms after the stimulus ('Post-stimulus') for all trials (thin grey lines) and the average over trials (mean ± s.d., thick black line).

(F) Post stimulus trial-average change in pixel values for $n = 3$ mice. Error bars are s.d. over trials. Average changes are not significantly different from zero (Wilcoxon signed-rank; mouse 1: n = 150 trials, W = 4853, p = 0.13; mouse 2: n = 25 trials, W = 133, p = 0.44; mouse 3: n = 25 trials, W = 152, p = 0.79).

bioRxiv preprint doi: https://doi.org/10.1101/2024.12.02.626335; this version posted March 13, 2025. The copyright holder for this preprint (which was not certified by peer review) is the author/funder, who has granted bioRxiv a license to display the preprint in perpetuity. It is made available under aCC-BY 4.0 International license.

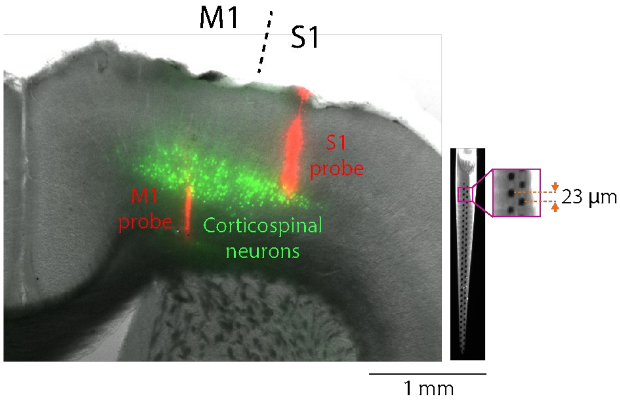

Figure 1 - figure supplement 2. Histological reconstruction of electrode placements in S1 and M1

780 **Left:** Example brain section, showing cortical labeling of fluorescent probe tracks (red). For this example, corticospinal neurons (green) were also labeled. **Right:** Example epifluorescence image of a dye-coated 64-channel probe. Inset shows a zoom-in view of the electrode (magenta square), 23 µm vertical spacing between channels in a horizontally staggered configuration. Total recording length from bottom-to-top channel: 1449 µm.

785

bioRxiv preprint doi: https://doi.org/10.1101/2024.12.02.626335; this version posted March 13, 2025. The copyright holder for this preprint (which was not certified by peer review) is the author/funder, who has granted bioRxiv a license to display the preprint in perpetuity. It is made available under aCC-BY 4.0 International license.

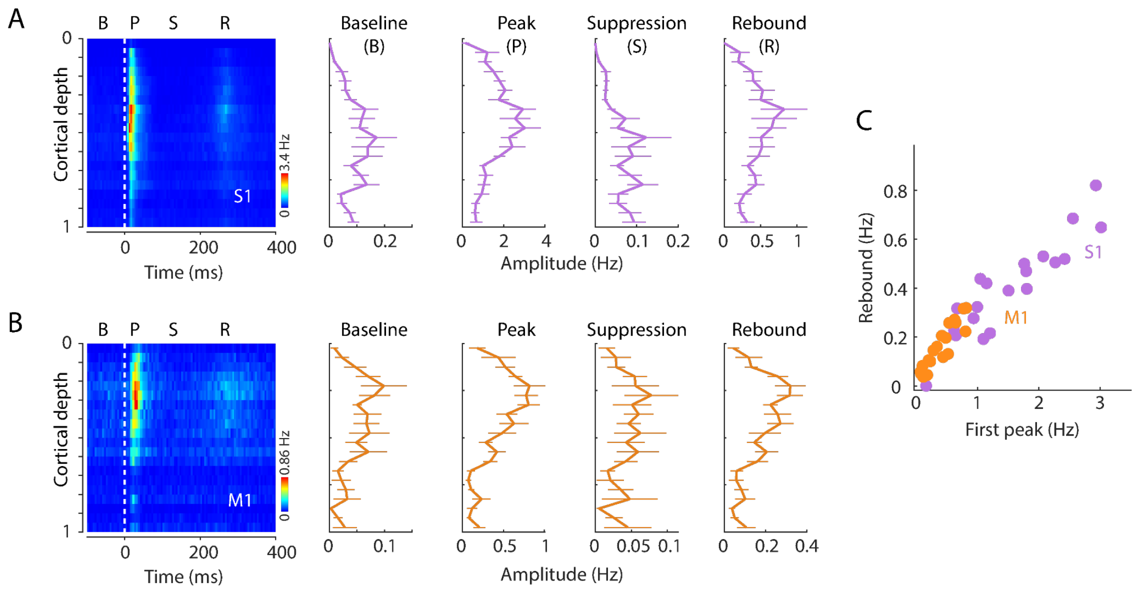

Figure 5 - figure supplement 1. Laminar profiles of activity across the triphasic response to hand stimulation

(**A**) Evoked spiking activity of units across the depth of S1 cortex. Plots show the overall amplitude (mean ± s.e.m, 11 recordings from 8 mice) during the baseline (B), initial peak (P), suppression (S), and rebound (R) intervals.

(**B**) Same, for M1 responses.

(**C**) S1 (purple) and M1 (orange) rebound versus initial peak response amplitudes in each of the 20 depth bins (Spearman's correlation index, S1: ρ =0.88, $p < 10^{-308}$; M1: ρ = 0.91, $p = 3 \times 10^{-6}$).

bioRxiv preprint doi: https://doi.org/10.1101/2024.12.02.626335; this version posted March 13, 2025. The copyright holder for this preprint (which was not certified by peer review) is the author/funder, who has granted bioRxiv a license to display the preprint in perpetuity. It is made available under aCC-BY 4.0 International license.

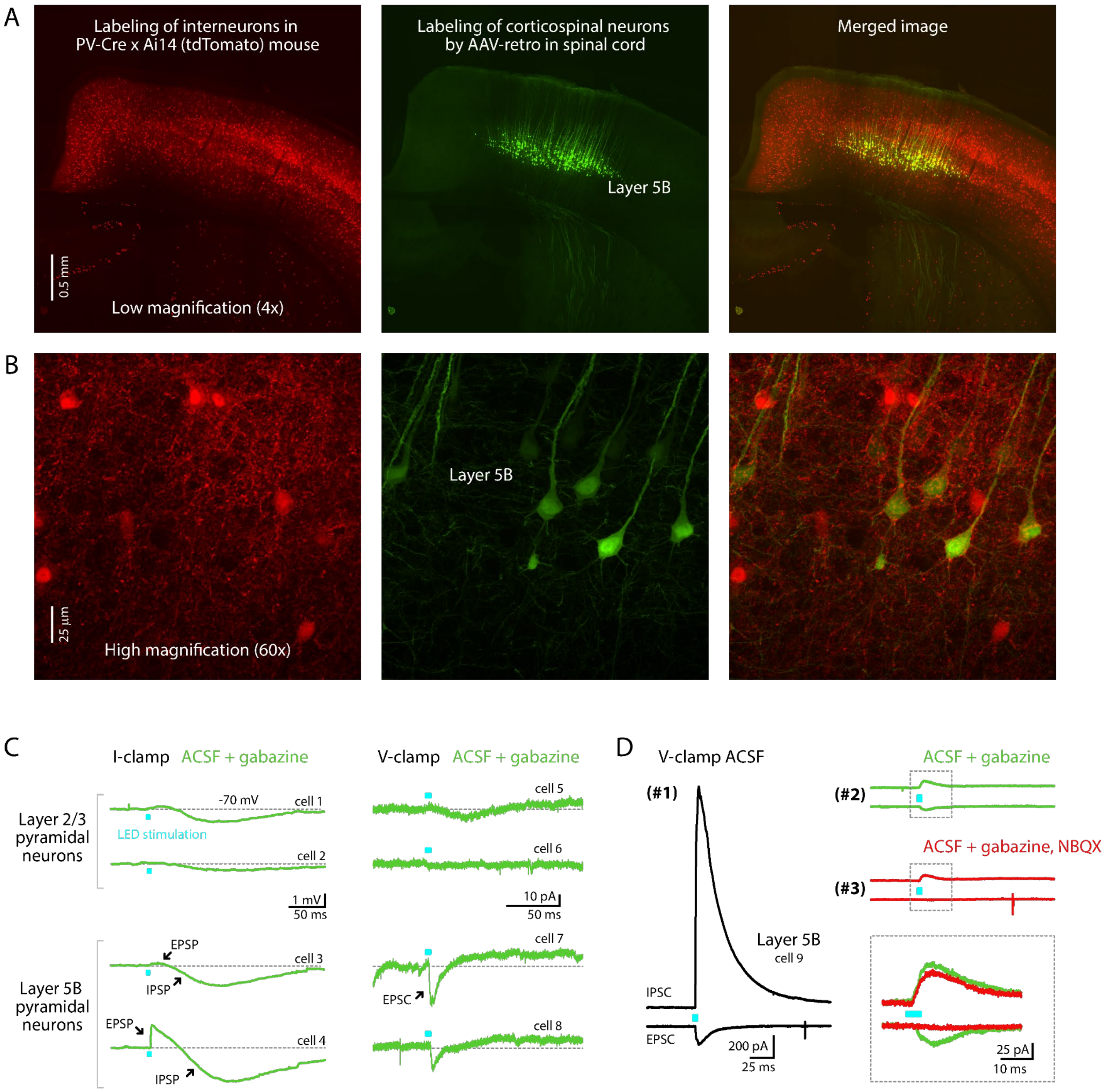

Figure 6 - figure supplement 1. Labeling patterns in PV-Cre mice

(**A**) Low-magnification confocal images showing the fluorescent labeling of cortical neurons in a PV-Cre x Ai14 (tdTomato-reporter) mouse **(left)** and corticospinal neurons (green) labeled retrogradely from the spinal cord **(middle)**, with a merged image **(right)**.

(**B**) High-magnification images from layer 5B of the same sections. Images are representative of results from 3 mice, showing no co-labeling; specifically, in each mouse 0% (0 of 50) of the tdTomato-expressing cells were corticospinal neurons, and 0% (0 of 50) of the corticospinal neurons expressed tdTomato.

(C) Example traces from optogenetic-electrophysiological assessment of possible excitatory responses evoked by photostimulation in acute brain slices from 2 PV-Cre x Ai32 (ChR2-reporter) mice. Responses of layer 2/3 and layer 5B pyramidal neurons to photostimulation of PV-expressing axons were recorded in the presence of gabazine (10 µM; GABA-A receptor

bioRxiv preprint doi: https://doi.org/10.1101/2024.12.02.626335; this version posted March 13, 2025. The copyright holder for this preprint (which was not certified by peer review) is the author/funder, who has granted bioRxiv a license to display the preprint in perpetuity. It is made available under aCC-BY 4.0 International license.

antagonist) in current-clamp (potassium-based internal solution) mode **(left)** or voltage clamp (cesium-based internal solution) mode **(right)**.

(D) Example traces show excitatory and inhibitory responses of a single layer 5B neuron recorded at -70 mV **(bottom traces; EPSCs)** and 10 mV **(upper traces; IPSCs)**. Responses were recorded first **(#1)** in control conditions with plain artificial cerebrospinal fluid (ACSF), then **(#2)** in the presence of gabazine, and finally **(#3)** after also adding NBQX (10 μM; AMPA receptor antagonist). Inset shows a close-up view on the area designated by the dashed square, indicating that a small (~25 pA) NBQX-sensitive inward (i.e., excitatory) current.

bioRxiv preprint doi: https://doi.org/10.1101/2024.12.02.626335; this version posted March 13, 2025. The copyright holder for this preprint (which was not certified by peer review) is the author/funder, who has granted bioRxiv a license to display the preprint in perpetuity. It is made available under aCC-BY 4.0 International license.

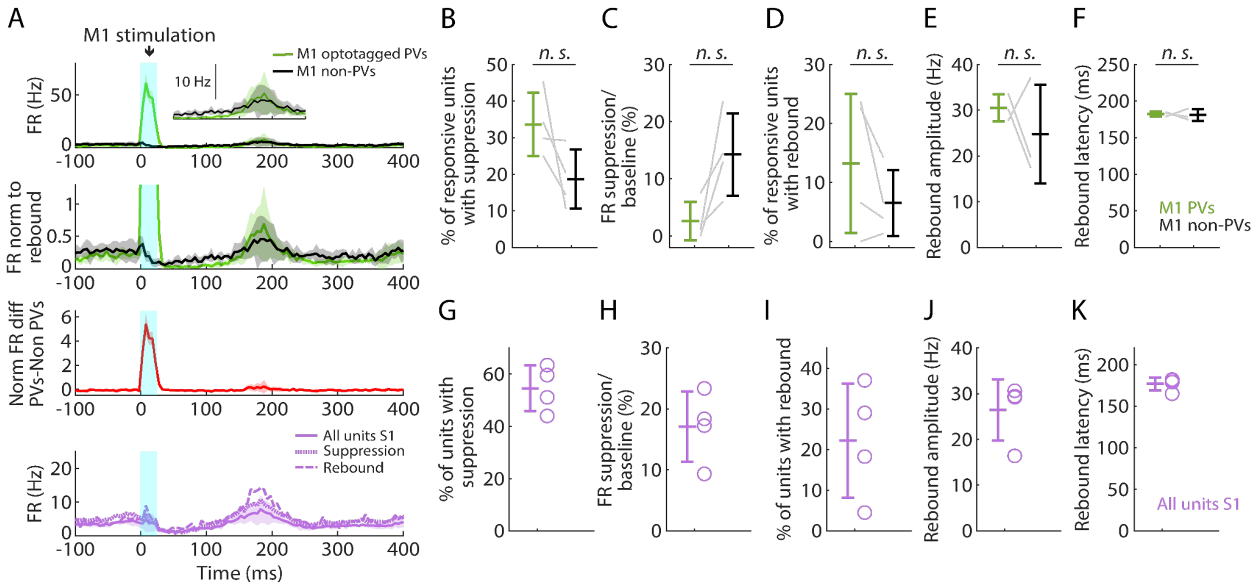

Figure 7 - figure supplement 1. Selective activation of PV neurons in M1 generates suppression and rebound

(A) Top plot: Grand average (mean ± s.d.) PSTHs for opto-tagged PV and non-PV units in M1 across recordings (4 recordings from 3 mice), aligned to the onset of focal M1 photostimulation (1 mW at the fiber tip, 105 µm core diameter). Inset on the right shows the rebound segment of the same data on a magnified y-axis scale. **2nd plot:** Grand average PSTHs normalized to the post peak rebound amplitude. **3rd plot:** Average difference of PV minus non-PV post peak rebound-normalized PSTHs. **4th plot:** Grand average PSTH for all S1 units, and for units with suppression or rebound.

(B) Percentage of PV and non-PV units with significant suppression (in the time window 110 to 170 ms) compared to pre-stimulus baseline (Paired samples t-test, t_3 =2.08, p = 0.13).

(C) Suppression as a percentage of baseline firing rate amplitude for PV and non-PV units in B (t_3 =-2.84, p = 0.07).

(D) Percentage of PV and non-PV units with significant rebound compared to pre-stimulus baseline (t_3 = 1.67, p = 0.19).

(E) Rebound amplitude for PV and non-PV units in D (t_2 = 0.76, p = 0.53).

(F) Rebound latency for PV and non-PV units in D (t_2 = 0.23, p = 0.82).

(G-K) Same as B-F but for all units recorded on the S1 probe during M1 photostimulation.

bioRxiv preprint doi: https://doi.org/10.1101/2024.12.02.626335; this version posted March 13, 2025. The copyright holder for this preprint (which was not certified by peer review) is the author/funder, who has granted bioRxiv a license to display the preprint in perpetuity. It is made available under aCC-BY 4.0 International license.

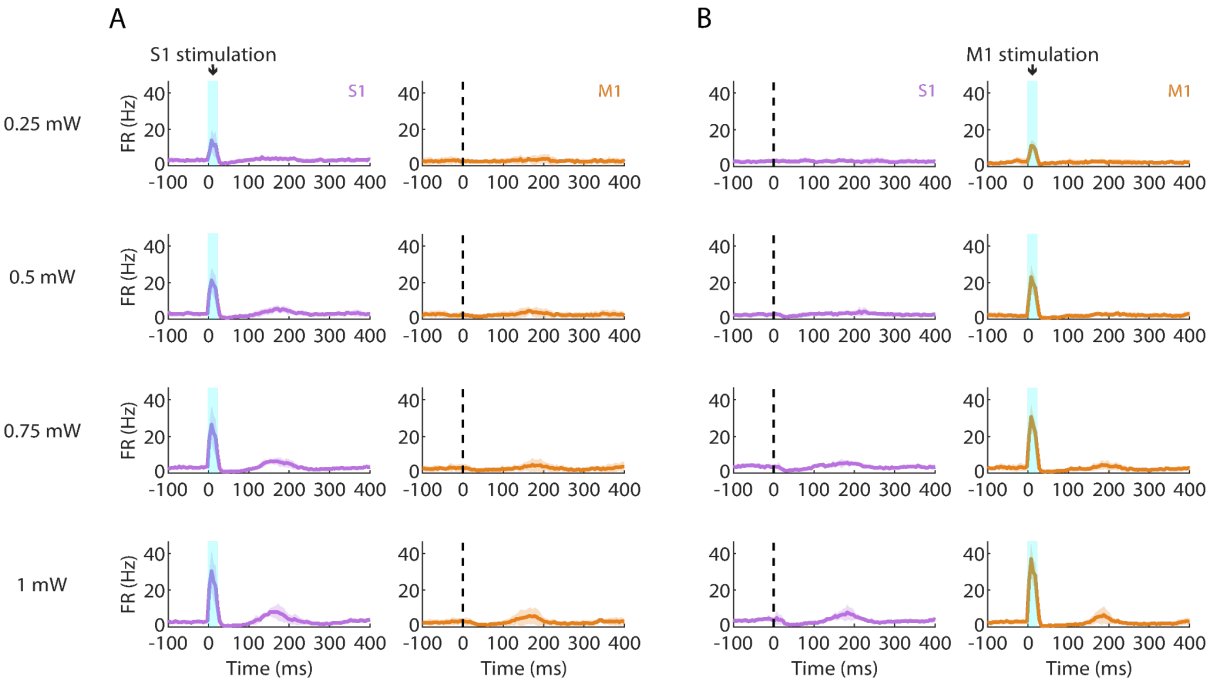

Figure 7 - figure supplement 2. Suppression and rebound induced by PV activation scale with photostimulation intensity.

(A) Left: Grand average (mean ± s.d.) PSTHs for S1 activity across recordings (4 recordings from 3 mice) during focal PV activation at a range of photostimulus intensities (0.25-1 mW at the fiber tip, 105 µm core diameter). **Right:** Simultaneous M1 recorded activity.

(B) Same as A but during M1 photostimulation.
