# Supplementary material for: Cortical dynamics in hand/forelimb S1 and M1 evoked by brief photostimulation of the mouse’s hand
Source: bioRxiv. 2024 Dec 2:2024.12.02.626335. Preprint. [Version 1] doi: 10.1101/2024.12.02.626335 (PMC11642753; doi:10.1101/2024.12.02.626335)
Supplement: Supplementary file 1 [file NIHPP2024.12.02.626335v1-supplement-1.pdf]

bioRxiv preprint doi: https://doi.org/10.1101/2024.12.02.626335; this version posted December 4, 2024. The copyright holder for this preprint (which was not certified by peer review) is the author/funder, who has granted bioRxiv a license to display the preprint in perpetuity. It is made available under aCC-BY 4.0 International license.

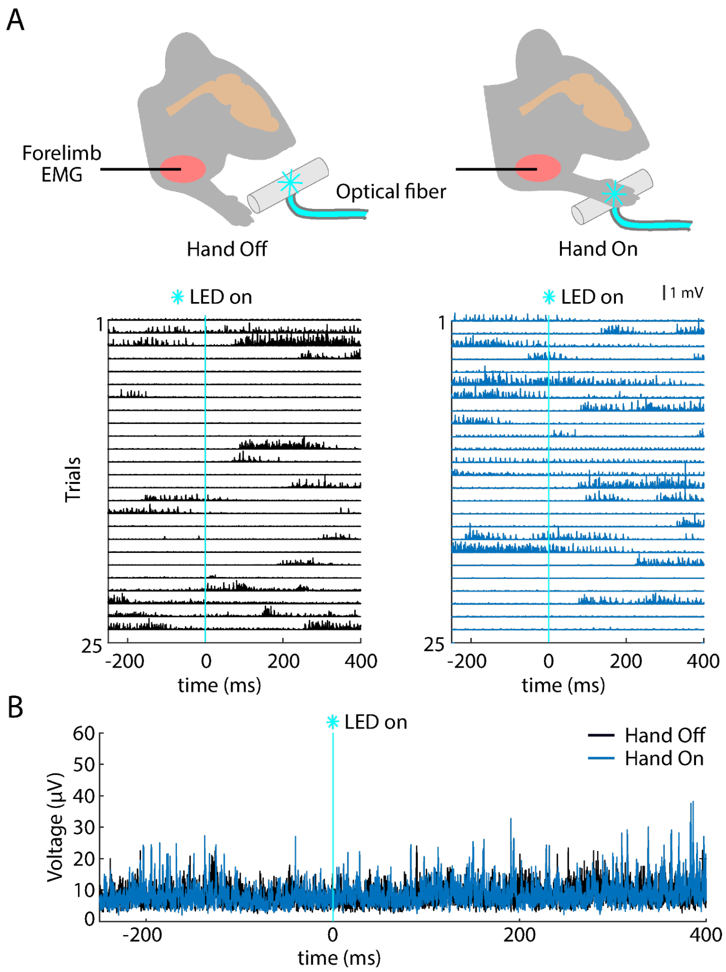

Supplementary Fig. S1. Optogenetic stimulation of mechanosensory afferents in the mouse's hand does not evoke forelimb movements

(A) Top: Schematic depicts EMG recording in biceps of an awake head-fixed mouse during flashes of blue LED light delivered to a holding bar when the hand was resting on (right, blue) or off (left, black) the bar. **Bottom:** EMG recording traces across trials aligned to the blue LED pulses while the mouse's hand was resting on or off the bar. Note the lack of consistent, time-locked changes in EMG amplitude following LED onset.

(B) Median stimulus-evoked EMG responses in biceps (25 trials) to blue LED pulses while the hand was resting on or off the light-delivery bar.

bioRxiv preprint doi: https://doi.org/10.1101/2024.12.02.626335; this version posted December 4, 2024. The copyright holder for this preprint (which was not certified by peer review) is the author/funder, who has granted bioRxiv a license to display the preprint in perpetuity. It is made available under aCC-BY 4.0 International license.

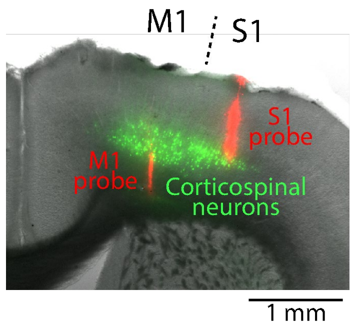

Supplementary Fig. S2. Histological reconstruction of electrode placements in S1 and M1
Example brain section, showing cortical labeling of fluorescent probe tracks (red). For this example, corticospinal neurons (green) were also labeled.

bioRxiv preprint doi: https://doi.org/10.1101/2024.12.02.626335; this version posted December 4, 2024. The copyright holder for this preprint (which was not certified by peer review) is the author/funder, who has granted bioRxiv a license to display the preprint in perpetuity. It is made available under aCC-BY 4.0 International license.

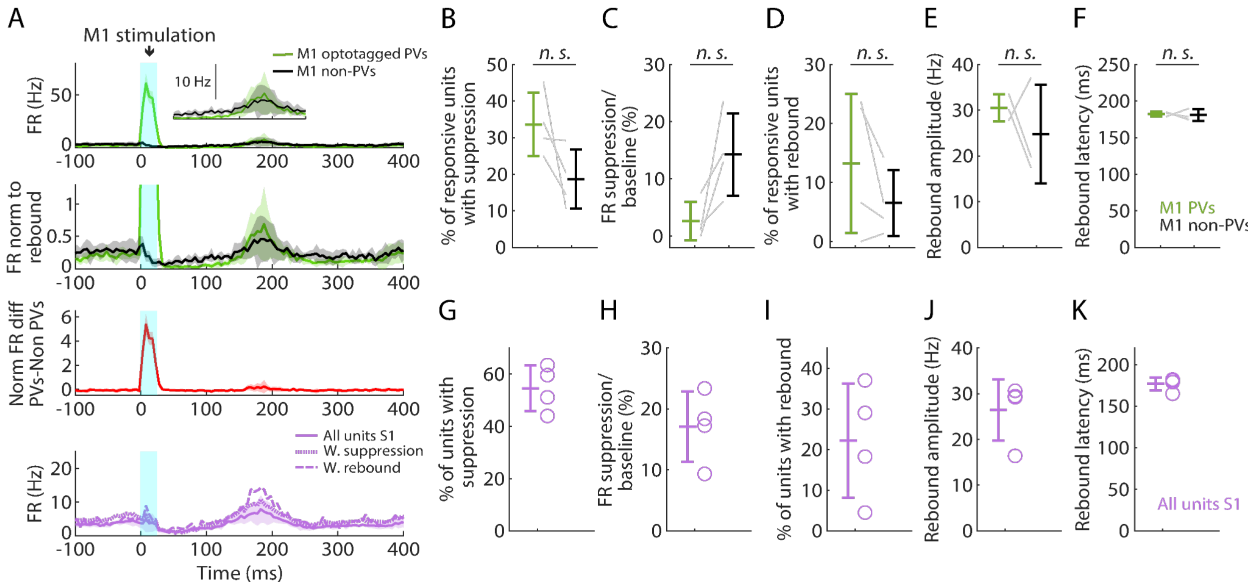

Supplementary Fig. S3. Selective activation of PV neurons in M1 generates suppression and rebound

(A) Top plot: Grand average (mean ± s.d.) PSTHs for opto-tagged PV and non-PV units in M1 across recordings (4 recordings from 3 mice), aligned to the onset of focal M1 photostimulation (1 mW at the fiber tip, 105 µm core diameter). Inset on the right shows the rebound segment of the same data on a magnified y-axis scale. **2nd plot:** Grand average PSTHs normalized to the post peak rebound amplitude. **3rd plot:** Average difference of PV minus non-PV post peak rebound-normalized PSTHs. **4th plot:** Grand average PSTH for all S1 units, and for units with suppression or rebound.

(B) Percentage of PV and non-PV units with significant suppression (in the time window 110 to 170 ms) compared to pre-stimulus baseline (Paired samples t-test, t_3 =2.08, p = 0.13).

(C) Suppression as a percentage of baseline firing rate amplitude for PV and non-PV units in B (t_3 =-2.84, p = 0.07).

(D) Percentage of PV and non-PV units with significant rebound compared to pre-stimulus baseline (t_3 = 1.67, p = 0.19).

(E) Rebound amplitude for PV and non-PV units in D (t_2 = 0.76, p = 0.53).

(F) Rebound latency for PV and non-PV units in D (t_2 = 0.23, p = 0.82).

(G-K) Same as B-F but for all units recorded on the S1 probe during M1 photostimulation.

bioRxiv preprint doi: https://doi.org/10.1101/2024.12.02.626335; this version posted December 4, 2024. The copyright holder for this preprint (which was not certified by peer review) is the author/funder, who has granted bioRxiv a license to display the preprint in perpetuity. It is made available under aCC-BY 4.0 International license.

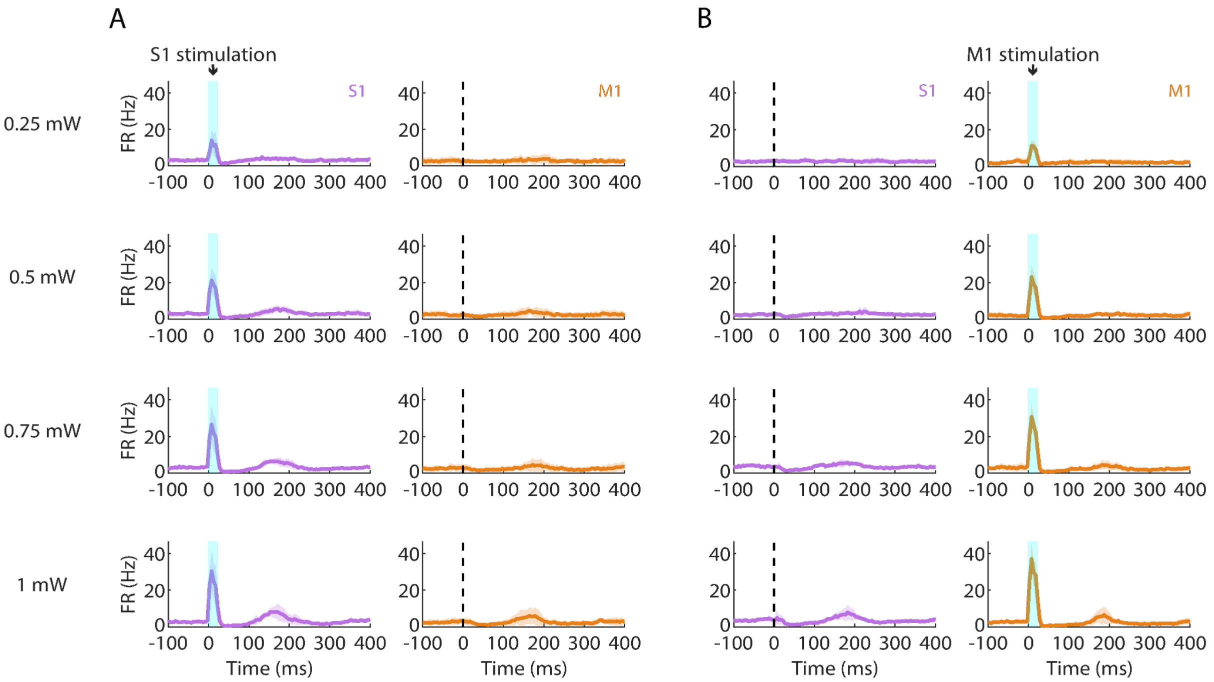

Supplementary Fig. S4. Suppression and rebound induced by PV activation scale with photostimulation intensity.

(A) Left: Grand average (mean ± s.d.) PSTHs for S1 activity across recordings (4 recordings from 3 mice) during focal PV activation at a range of photostimulus intensities (0.25-1 mW at the fiber tip, 105 µm core diameter). **Right:** Simultaneous M1 recorded activity.

(B) Same as A but during M1 photostimulation.
